# Effect of a CO_2_ Laser on the Inhibition of Root Surface Caries Adjacent to Restorations of Glass Ionomer Cement or Composite Resin: An *In Vitro* Study

**DOI:** 10.1155/2015/298575

**Published:** 2015-08-12

**Authors:** L. C. Daniel, F. C. Araújo, B. R. Zancopé, F. S. Hanashiro, M. Nobre-dos-Santos, M. N. Youssef, W. C. Souza-Zaroni

**Affiliations:** ^1^School of Dentistry, Cruzeiro do Sul University (UNICSUL), Avenida Doutor Ussiel Cirilo 225, 08060-070 São Paulo, SP, Brazil; ^2^Department of Pediatric Dentistry, Faculty of Dentistry of Piracicaba, State University of Campinas (UNICAMP), Avenida Limeira 901, P.O. Box 52, 13414-903 Piracicaba, SP, Brazil; ^3^Department of Restorative Dentistry, School of Dentistry, University of São Paulo (USP), Avenida Professor Lineu Prestes 2227, Cidade Universitária, 05508-900 São Paulo, SP, Brazil

## Abstract

This study investigated the effect of CO_2_ laser irradiation on the inhibition of secondary caries on root surfaces adjacent to glass ionomer cement (GIC) or composite resin (CR) restorations. 40 dental blocks were divided into 4 groups: G1 (negative control): cavity preparation + adhesive restoration with CR; G2: (positive control) cavity preparation + GIC restoration; G3: equal to group 1 + CO_2_ laser with 6 J/cm^2^; G4: equal to group 2 + CO_2_ laser. The blocks were submitted to thermal and pH cycling. Dental demineralization around restorations was quantified using microhardness analyses and Light-Induced Fluorescence (QLF). The groups showed no significant differences in mineral loss at depths between 20 *μ*m and 40 *μ*m. At 60 *μ*m, G2 and G3 ≠ G1, but G4 = G1, G2 and G3. At 80 *μ*m, G4 ≠ G1, and at 100 *μ*m, G4 = G2 = G1. At 140 and 220 *μ*m, G2, G3, and G4 = G1. The averages obtained using QFL in groups 1, 2, 3, and 4 were 0.637, 0.162, 0.095, and 0.048, respectively. QLF and microhardness analyses showed that CO_2_ laser irradiation reduced mineral loss around the CR restorations but that it did not increase the anticariogenic effect of GIC restorations.

## 1. Introduction

Clinical studies showed that secondary caries lesions are the most common cause for restoration replacement [1–4]. These lesions are even more serious when the restorative procedure is performed on root surfaces because of the patient's difficulty in performing oral hygiene correctly and the difficulty in isolating the operative field and accessing the lesion [5]. Therefore, new methods, such as irradiation of tooth surfaces using different types of lasers, were studied to control the recurrence of caries in this region.

Root caries are more frequent in elderly patients [6–8], and these lesions are generally related to a variety of factors, such as decreased salivary flow, xerostomic medication, periodontal disease, and motor difficulties, which often prevent proper cleaning.

The root surface is rougher than enamel, which facilitates the formation of biofilm in the absence of proper oral hygiene. Carious lesions progress rapidly in this area because of the difference in chemical composition and structure of the mineral tissues of the teeth. The cementum and dentin have a critical pH value for dissolution of approximately 6.7 [9], but the critical pH for enamel is 5.5.

Konishi et al. [10] demonstrated that caries removal using CO_2_ laser irradiation produced cavity walls around restorations that were more resistant to caries than those produced using conventional mechanical removal. Klein et al. [11] also showed that irradiation of the enamel around composite resin restorations using a CO_2_ laser inhibited demineralization of the enamel adjacent to the cavity preparation.

Souza-Zaroni et al. [12] showed that irradiation of the root surface using CO_2_ laser power densities of approximately 4.0 to 6.0 J/cm^2^ promoted the inhibition of demineralization on the root surface. Similarly, de Melo et al. [13] observed that a CO_2_ laser effectively inhibited the demineralization of root surfaces adjacent to composite restorations at energy densities of 5.0 and 6.0 J/cm^2^.

However, existing studies in the literature have not focused on the effect of irradiation of cavosurface angles using a CO_2_ laser on cavity preparations restored with known anticariogenic materials, such as glass ionomer cement.

## 2. Materials and Methods

### 2.1. Experimental Design

This study was conducted after being approved by the Research Ethics Committee of Cruzeiro do Sul University (CE/UCS-135/2012). The factors studied were the treatment conducted on the cavosurface margins of root cavities with or without irradiation using a CO_2_ laser and the restorative material (glass ionomer cement or composite resin).

Cavity preparations in group 1 (negative control) were performed on ten tooth blocks using a diamond bur number 2294 (KG Sorensen, Barueri, São Paulo, Brazil) in a high-speed turbine. The blocks were submitted to phosphoric acid etching and an adhesive system was applied. The blocks were restored with a resin composite. Cavities in group 2 (positive control) were prepared on ten tooth blocks using a diamond bur mounted in a high-speed turbine. The cavities were restored with glass ionomer cement. Ten tooth blocks with cavity preparations were performed in group 3 using a diamond bur mounted in a high-speed turbine. The cavosurface margins were submitted to surface treatment with a CO_2_ laser at an energy density of 6 J/cm^2^. The margins were etched with phosphoric acid, and an adhesive system was applied. The margins were restored with resin composite. Cavities in group 4 were prepared on ten tooth blocks using a diamond bur mounted in a high-speed turbine, and the cavosurface margins were surface-treated with irradiation using a CO_2_ laser at an energy density of 6 J/cm^2^. The cavities were restored with glass ionomer cement.

### 2.2. Preparation of Specimens

Bovine incisors were stored in a 0.1% thymol solution for a minimum of 30 days after cleaning with periodontal curettes, followed by prophylaxis with a Robinson brush at low speed using aluminum paste (5 *μ*m) for 30 s and two 30-s ultrasound baths. The teeth were examined under a stereoscopic loupe at 10x magnification. Incisors with cracks or structural abnormalities were discarded, and the remaining teeth were kept in a humid medium at 4°C until use.

The teeth were fixed in a cutting machine to obtain the root surface specimens. The coronal portion 1 mm beyond the cement-enamel junction was removed and discarded. The root portion of each tooth was longitudinally sectioned to obtain two specimens (one from the buccal and one from the lingual surface). The sections were identified to avoid the use of two specimens from the same tooth in the same group.

### 2.3. Cavity Preparation

A number 2294 cylindrical diamond bur (KG Sorensen, Barueri, Brazil) in a high-speed turbine with air-water spray was used. A standard cavity with a diameter of 1.7 ± 1 mm and depth of 1.5 mm was prepared.

### 2.4. Restorative Procedure

Prophylaxis was performed after the cavity preparations in all the dental root blocks using a pumice stone paste (SS White) and distilled and deionized water. The cavities were washed with a water and air spray and air dried.

Preparations in groups 1 and 3 were restored with a resin composite (Filtek Z 250, 3 M ESPE, St. Paul, MN, USA) using 37% phosphoric acid etching and Single Bond 2 adhesive (3 M ESPE, St. Paul, MN, USA) before insertion of the resin composite. The preparations in groups 2 and 4 were restored using conventional glass ionomer cement (GIC) (Ketac Fil Plus, 3 M ESPE, St. Paul, MN) with the aid of a Centrix syringe to avoid the inclusion of bubbles within the restorative material. Subsequently, the GIC was covered with a polyester strip, and a glass slide and weight (500 g) were placed on top for 7 min to standardize the restorations.

The materials were manipulated, and the cavities were restored in accordance with the manufacturer's instructions. The dental blocks were stored for 24 h at 37°C in a 100% humid environment at the conclusion of the restorative procedure, and the blocks were polished using a sequence of aluminum oxide disks (Sof-Lex, 3 M ESPE, St. Paul, MN, USA).

### 2.5. Quantification of Light-Induced Fluorescence (QLF)

The prevention of caries was also quantified using light-induced fluorescence (QLF).

The area of the root surface that would receive the proposed treatments in the area around the restorations was delimited (cavosurface margins of the preparations made on the root surface). Each specimen was completely covered with acid-resistant varnish (colorless cosmetic nail polish), except a 4 × 4 mm area of exposed root (window) whose center contained the cavity that was restored with composite resin or glass ionomer cement, which was 1.6 mm in diameter.

Images were taken of all of samples, first with only the restorations (baseline) and again after thermal and pH cycling (final). Images of all samples were captured using the Inspektor Pro intraoral fluorescence camera (Inspektor Dental Care BV, Amsterdam, The Netherlands). The samples were exposed to blue-violet light, which peaked in intensity at 404 nm. The emitted fluorescence of the dental structure was observed through a high-pass yellow filter (*λ* ≥ 520 nm) and recorded by the camera device. The camera was fixed to a support, and the sample surface remained perpendicular to the device hand piece, where the blue light source and the fluorescence camera were located. The distance between the sample surface and the apparatus hand piece was adjusted to obtain the best focus.

Samples that were stored in flasks containing distilled water had their dentin surface dried with absorbent paper before fluorescence analyses.

Samples were placed on a plastic disk, and the images were obtained in a dark room. An image was captured for each sample to enable the subsequent evaluations of fluorescence loss.

The images were examined using Inspektor Pro version 2.0.0.32 software (Inspektor Dental Care BV, Amsterdam, The Netherlands), and the values of Delta *Q* (Δ*Q*) were calculated assuming a 5% threshold. Therefore, variations in fluorescence between the sound and demineralized dentin that were smaller than 5% were disregarded. A single examiner performed all analyses using a set of standard rules for image analysis to ensure the reliability of the measurements [14].

### 2.6. Cavosurface Angle Treatment Using a Pulsed CO_2_ Laser

Irradiation of the cavosurface margins of root surface preparations was performed using a pulsed CO_2_ laser at a wavelength of 10.6 *μ*m (UM-L30, Union Medical Engineering Co., Yangju-si, Gyeonggi-Do, Korea—FAPESP CEPID/CEPOF Project, process number 98/14270-8). The selection of the irradiation parameters was based on a previous study by Souza-Zaroni et al. [12], which was also conducted on root surfaces.

The specimens were irradiated at a fixed repetition rate of 50 Hz with a pulse duration of 10 ms on and 10 ms off, and a beam diameter of 0.3 mm. The power was 0.8 W. The power set on the appliance was confirmed using a power meter (Scientech 373 Model: 37-3002, Scientech, Inc., Boulder, CO, USA), and the mean power measured was 0.42 W. The energy density under these irradiation conditions was approximately 6 J/cm^2^. Irradiation was performed with the active point of the laser perpendicular to the tooth surface at a distance of 1 cm for a period of 10 s, and the speed of lasing movement was 2 mm/s.

### 2.7. Thermal Cycling

The main objective of this procedure was to expose the restorative material to a high thermal challenge. All the groups in the thermal cycling process were stored in tulle bundles with each containing one group, which were submitted to 1,000 cycles. Each cycle consisted of immersion in distilled and deionized water for 60 s at a temperature of 5 ± 1°C and a temperature of 55 ± 1°C for an additional 60 s. Temperatures in the thermal cycling machine remained constant.

### 2.8. pH Cycling

We used the model described by Kawasaki and Featherstone [15] and modified by Souza-Zaroni et al. [12] to perform pH cycling. The pH cycling was performed for 5 days. The root specimens were immersed for 4 h/day in the demineralizing solution and the remineralizing solution for approximately 20 h/day. The specimens were washed for 10 s with deionized water twice daily (before and after immersion in the demineralizing solution) and dried with absorbent paper during this period. The specimens remained in the remineralizing solution for 2 days (corresponding to the days of the weekend) after the fifth day.

The specimens were kept individually in the demineralizing solution containing 2.0 mmol/L Ca and 2.0 mmol/L P in 75 mmol/L acetate buffer, pH 4.8. The remineralizing solution contained 1.5 mmol/L Ca, 0.9 mmol/L P, and 150 mmol/L KCl in 20 mmol/L Tris buffer, pH 7.0. The chemical composition of this solution was near the level of saturation of the apatite minerals found in saliva, and it was similar to the solution by Ten Cate and Duijsters [16]. Both solutions contained thymol crystals to prevent bacterial growth, and solutions were prepared using the same reagents as sources of calcium and phosphate. The quantities of 6.25 and 3.12 mL/mm^2^ of de- and remineralizing solutions were used, respectively, per treatment area. The specimens remained in an oven set at 37°C during the entire process, except the intervals of washing and alternating solutions. Specimens in the pH cycling were washed with jets of distilled and deionized water for 10 s, dried with absorbent paper, and kept in a closed and humid ambient environment with undercooling until they were prepared for microhardness analyses.

### 2.9. Cross-Sectional Microhardness Analysis

Specimens were sectioned in the center of the window of exposed dentin after the pH cycling using a precision cutting machine (Labcut, 1010, Extec, USA) and a diamond disk with a thickness of 0.3 mm and undercooling. One-half of each specimen was selected and positioned in the center of a semirigid plastic tube (sample cups). The specimen was embedded in an acrylic resin that was poured over it, and the internal (sectioned) part of the specimen was exposed. The plastic tubes were removed after resin polymerization, and the specimens were polished with water abrasive papers in a decreasing order of grit (numbers 600 and 1000) for 1 and 5 min, respectively. Felt disks and a 6 *μ*m diamond paste (2 min) and a 3 *μ*m diamond paste (4 min) were used after abrasive paper polishing. All specimens were submitted to an ultrasound bath of distilled and deionized water for 3 min between changes of abrasive papers and between the pastes/suspensions with common liquid detergent diluted in water for 5 min. The specimens were washed abundantly under distilled and deionized water and stored in a closed and humid environment with undercooling until use.

The microhardness test was performed to verify the presence or absence of demineralization on the root surface adjacent to the tooth/restoration interface.

The blocks were visualized with the aid of a monitor. Fourteen indentations were made on each dental block at different points on the root surface adjacent to the tooth/restoration interface of each tooth at a standardized distance for all of the restorations evaluated.

Analyses were performed using the microhardness tester HMV-2000 (Shimadzu Corporation, Kyoto, Japan) and the Knoop-type penetrator with a 5-g load and a 15-s application duration. Indentations were made longitudinally on the cut faces with the long axis of the diamond indenter parallel to the external portion of the root surface. The indentations were located 100 *μ*m and 3 mm from the tooth/restoration interface and were 20, 40, 60, 80, 100, 140, and 220 *μ*m in depth from the cavosurface margins (tooth external surface) in the direction of the pulp tissue (adapted from Klein et al. [11]).

### 2.10. Statistical Analysis

The SPSS program was used for data analyses. The presuppositions necessary for the analysis of variance (ANOVA) were verified. After ANOVA, multiple comparisons of the means were performed using the Games and Howell test at a 5% level of significance. This parametric test is indicated for deviations from normality and homogeneity. The Games and Howell test was performed for each pair of averages (“pairwise”) with the error set for each comparison.

## 3. Results

This work had two variation factors, the treatment performed on the cavosurface margins of the preparations on the root surface (with or without CO_2_ laser irradiation) and the material used to restore the cavities (glass ionomer cement or composite resin). The variation factors were analyzed using two response variables, the loss of fluorescence using the QLF technique, which is represented by the value of Delta *Q* (Delta *Q* = percentage loss of fluorescence per square millimeter), and the mineral loss values, which were calculated as the difference between the Knoop microhardness values of the healthy area (3 mm away from the tooth/restoration interface) and the microhardness values of the decayed area (100 *μ*m interface) at different depths from the surface of the tooth.

### 3.1. QLF

Table 1 presents the mean QLF values of the surface treatment factors. The negative control group was significantly different from the other groups and presented the lowest Delta *Q* value. The G3 and G4 groups were not statistically significantly different, but G3 was similar to G2. Higher Delta *Q* values were obtained for G2 than for the groups that received laser irradiation (G3 and G4), which demonstrates that the specimens in G2 lost less fluorescence.

### 3.2. Microhardness Analysis

Table 2 presents the mean mineral loss values of the surface treatment factors. The depths of 20 to 40 *μ*m were statistically similar in all groups. G2, G3, and G4 were similar at the depth of 60 *μ*m, but G4 was not significantly different from G1. However, G3 was higher than the G1 and G4 groups at the depth of 100 *μ*m, but it was similar to G1. The G2, G3, and G4 groups were similar but higher than G1 at the depths of 80, 140, and 220 *μ*m.

## 4. Discussion

Fluoride is a well-documented anticariogenic product in the literature. The mechanisms that characterize the cariostatic effects of fluoride include reduction of demineralization and increase of remineralization, but the mechanisms also include interference of film and plaque formation and the inhibition of bacterial growth and metabolism [17–19].

A wide variety of vehicles release fluoride into the oral cavity, including mouthwashes, toothpastes, and fluoride-releasing restorative materials [20–23]. Fluoride released from dental restorative materials affects caries formation through all the mechanisms mentioned above, which reduce or prevent demineralization and promote the remineralization of hard dental tissues [24].

There are various dental restorative materials available on the market that contain fluoride, including glass ionomer cement, resin-modified glass ionomer, polyacid-modified composite, composite resin, and amalgam alloys. These products vary in their ability to release fluoride because of their different matrixes and setting mechanisms. However, it is assumed that the antibacterial and cariostatic properties of these restorative materials are associated with the amount of fluoride released.

Our study demonstrated the effectiveness of conventional glass ionomer cement (GIC) to prevent the occurrence of secondary caries on root surfaces [21–23, 25, 26]. This material showed an inhibition rate of 92.46% in QLF analysis, and it was superior to all other experimental groups, including the group that was irradiated with the CO_2_ laser. However, the GIC group was always higher than the control group in microhardness analyses, but it did not differ from the laser-irradiated groups.

The detection of root surface demineralization using a QLF system was demonstrated previously in* in vitro* and* in vivo* studies [27, 28]. Van der Veen [28] reported that the QLF system detects root surface lesions that are at least 80 *μ*m deep.

The rate of inhibition in our study using the QLF system was higher than previously reported in the literature. Therefore, caries lesions in dentin and cementum root surfaces adjacent to Class V restorations were reduced by 54–63% (glass ionomer), 20–53% (resin-modified glass ionomer), or 14–35% (compomer), compared to nonfluorinated control materials [20, 26, 29–31].

GIC promotes the release of approximately 10–50 times more fluoride than fluoridated composites [32]. Materials, such as resin-modified glass ionomer (RMGI) and polyacid-modified resin composite (RCMP), have intermediate cariostatic effects [22, 25].

The assessment of the extent of the cariostatic effect on root surface by measuring the microhardness demonstrated the action of GIC to be 220 *μ*m. Hara et al. [33] found that a fluoride-containing composite and a compomer exhibited no cariostatic effect, but the extent of the cariostatic effect of conventional glass ionomer cement was observed to be 300 *μ*m and 150 *μ*m for the resin-modified glass ionomer.

The inhibition of demineralization around GIC restorations was not increased, but the CO_2_ laser promoted the cariostatic effect around cavities restored with conventional composite resin. The inhibition rate of this group was approximately 85% in the QLF analysis. This finding is greater than that of Gao et al. [34], who reported that the reducing of root surface demineralization using a *λ* 10.6 *μ*m CO_2_ laser at an energy density of 1.14 J/cm^2^ was 29.8%. Souza-Zaroni et al. [12] showed a reduction of demineralization of the root surface of 17.05 and 20.59% using a *λ* 10.6 *μ*m CO_2_ laser and energy densities of 5 and 6 J/cm^2^, respectively. De Melo et al. [13] reported that the highest rate of inhibition achieved around composite restorations was 29.21%.

The mechanism of laser irradiation interactions with dental tissues in the absence of fluoride is mainly related to the temperature rise after absorption. In general, irradiation of the dentin using a CO_2_ laser causes changes in both the mineral portion and the organic matrix. The carbonate content can be reduced or eliminated depending on the energy applied, and crystallinity can be increased [35, 36]. A reduction in collagen content, loss of water, and formation of bands of amorphous carbon were also observed [37]. However, the reduction of carbonate and changes in the phases of hydroxyapatite that occur between 600 and 900°C are related to a decrease in the solubility of teeth after laser irradiation [35, 36, 38]. These tissue modifications are related to temperature, and not all laser irradiation conditions cause heating in the range that precisely positively changes the tissue to make it more resistant to decay.

Favorable results using the CO_2_ laser were observed by other authors who measured the dissolution of calcium and phosphorus [39–41] and lesion depth [34]. However, most previous studies were conducted using a CO_2_ laser emitting in a continuous mode, which is not the safer condition for the irradiation of vital teeth [35]. Only Esteves-Oliveira et al. [42] used a CO_2_ laser with or without APF gel at energy densities of 8 and 11 J/cm^2^ to achieve favorable results with the association of 11 J/cm^2^ laser irradiation and APF gel. These results also suggest a synergistic effect of this association, but the inhibition rate was approximately 15%.

Several recent studies [43–45] failed to find any increase in the acid resistance of dentin after irradiation with CO_2_ laser. Therefore, the positive results in this study should provide a stimulus to study new associations between the use of laser irradiation and fluoride-releasing materials to enable the optimization of additional tools for the prevention of root caries.

## 5. Conclusion

CO_2_ laser with *λ* = 10.6 *μ*m was effective in the reduction of mineral loss around the composite resin restorations on root surfaces; however it did not increase the anticariogenic effect of glass ionomer restorations. In this way, CO_2_ laser can be a resource in the prevention of secondary caries lesions on elderly population.

## Figures and Tables

**Table 1:** QF means for all groups tested.

Groups	Means	SD	% inhibition
G1: negative control	−0.637^a^	±0.33	—
G2: GIC	−0.048^c^	±0.04	92.46
G3: laser	−0.095^bc^	±0.09	85.09
G4: GIC + laser	−0.162^b^	±0.08	74.57

Similar letters indicate statistical similarity. SD: standard deviation.

**Table 2:** Microhardness analysis means (M) and standard deviations (SD) for all groups tested (similar letters indicate statistical similarity).

Groups	Depths													
	20 *μ*m		40 *μ*m		60 *μ*m		80 *μ*m		100 *μ*m		140 *μ*m		220 *μ*m	
	M	SD	M	SD	M	SD	M	SD	M	SD	M	SD	M	SD
G1: control	17.69	4.04	18.33	3.88	21.54	4.47^b^	24.42	5.43^b^	24.88	5.35^c^	26.06	5.85^b^	28.12	7.48^b^
G2: GIC	15.62	7.06	16.47	6.26	14.62	3.56^a^	14.79	3.90^a^	15.61	4.69^ab^	14.90	4.42^a^	14.34	4.23^a^
G3: laser	12.83	5.62	13.28	4.11	12.71	3.07^a^	12.67	4.18^a^	11.90	2.56^a^	12.51	3.27^a^	13.44	4.24^a^
G4: GIC + laser	14.15	5.22	16.00	6.86	12.21	3.43^ab^	17.08	7.38^a^	18.93	7.56^bc^	18.40	7.01^a^	17.66	8.22^a^
